# Case Report: Smith-Magenis syndrome with bilateral high-grade vesicoureteral reflux, hypothyroidism, and seizure in infancy

**DOI:** 10.3389/fped.2026.1906694

**Published:** 2026-10-15

**Authors:** Yun Cheng, Xunwei Liu, Lingyun Yang, Jinwen Xu

**Affiliations:** Department of Pediatric Nephrology, Rheumatology and Immunology, Children’s Hospital of Jiangnan University (Wuxi Children’s Hospital), Wuxi, Jiangsu, China

**Keywords:** 17p11.2 microdeletion, hypothyroidism, RAI1, reflux nephropathy, seizure, Smith-Magenis syndrome, vesicoureteral reflux

## Abstract

Smith-Magenis syndrome (SMS) is a rare neurodevelopmental and multisystem disorder caused mainly by 17p11.2 deletions involving RAI1. Because the characteristic behavioral and sleep phenotype may not yet be apparent, diagnosis in infancy can be challenging. We report a 9-month-old boy with recurrent febrile urinary tract infection, bilateral hydronephrosis with ureteral dilatation, hypothyroidism, global developmental delay, hypotonia, brachydactyly, dysmorphic features, phimosis, and short penis. Voiding cystourethrography demonstrated bilateral grade IV vesicoureteral reflux (VUR). Renal scintigraphy showed focal right renal cortical defects; DTPA effective renal plasma flow was 180.33 mL/min in the left kidney and 133.77 mL/min in the right kidney, corresponding to split function of 57.4% and 42.6%. Trio whole-exome sequencing with copy-number-variant analysis identified a *de novo* 3.63-Mb deletion at 17p11.2 encompassing RAI1, confirming SMS; a maternally inherited heterozygous DUOX2 likely pathogenic variant was also detected, but its contribution to hypothyroidism remains unproven. Infection improved with antibiotics, levothyroxine was resumed, and amoxicillin/clavulanate prophylaxis was initiated. Surgery for phimosis was postponed after an afebrile sleep-related seizure. Follow-up showed improved renal indices, no recurrent urinary tract infection, normalized thyroid function, and no definite abnormality on video-electroencephalography, although developmental delay persisted and brief seizure-like ocular events remained under evaluation. This case shows that high-grade bilateral VUR with recurrent infection and a syndromic neurodevelopmental phenotype can be an early presentation of SMS and warrants copy-number analysis, renal protection, and coordinated endocrine, neurologic, developmental, and urologic follow-up.

## 1 Introduction

Smith-Magenis syndrome (SMS; OMIM #182290) is a rare neurodevelopmental disorder characterized by developmental delay or intellectual disability, infantile hypotonia, distinctive craniofacial and skeletal features, sleep disturbance, behavioral dysregulation, and variable multisystem involvement. Most affected individuals have a heterozygous 17p11.2 deletion including RAI1; a smaller proportion have a pathogenic intragenic RAI1 variant (1–6). Because the characteristic behavioral phenotype often becomes evident only after infancy, early recognition depends on the combined neurodevelopmental, dysmorphic, and congenital-anomaly pattern.

RAI1 haploinsufficiency explains the core SMS phenotype, whereas genes elsewhere in the deleted interval may contribute to structural and organ-system manifestations. Genitourinary anomalies are reported in approximately 15%–35% of individuals with a 17p11.2 deletion and have not been reported in comparable cohorts with intragenic RAI1 variants (1, 7). Reported abnormalities include collecting-system duplication, renal agenesis or ectopia, ureterovesical obstruction or malposition, and genital anomalies.

Published evidence specifically linking SMS with VUR remains limited. The closest prior comparator was an 8-year-old boy with bilateral grade IV VUR who required bilateral ureteroplasty (8). We describe an infant with genetically confirmed SMS, recurrent febrile urinary tract infection, bilateral grade IV VUR, early right renal involvement, hypothyroidism, and a seizure-like presentation, with quantitative renal scintigraphy and short-term multidisciplinary follow-up.

## 2 Case description

### 2.1 Patient information and clinical findings

A 9-month-old boy was admitted to our hospital in May 2025 because of fever for 1 day with increased urine leukocytes. The highest temperature was 39.2 °C and was accompanied by chills. There was no gross hematuria, cloudy urine, or crying during urination. Urinalysis in the emergency department showed marked pyuria, and the patient received one dose of intravenous ceftriaxone before admission (Table 1).

**Table 1:** Clinical timeline and key investigations.

Time/age	Clinical event	Key findings	Clinical meaning
Birth/neonatal period	Cesarean delivery at 35 + 5 weeks; birth weight 2,700 g; Apgar scores 10 at 1 and 5 min; no NICU admission or respiratory support.	Heel-prick screen suspicious positive; TSH 8.58 μIU/mL and free T4 5.71 pmol/L; levothyroxine started promptly.	Perinatal factors did not explain the multisystem phenotype; congenital hypothyroidism was recognized early.
2025-02-23	Hospitalization for UTI with sepsis, bilateral hydronephrosis with ureteral dilatation, hypothyroidism, oral candidiasis, anemia, and abnormal brain development.	WBC 21.46 × 10⁹/L; CRP 120.6 mg/L; urine protein (+), leukocyte esterase (++), WBC 512.67/μL; lactate 7.4 mmol/L; procalcitonin 10.2 ng/mL.	Severe infection and structural urinary tract disease were present before the current admission.
2025-02-23	Renal, biochemical, and thyroid testing during the earlier hospitalization.	Potassium 3.74 mmol/L; sodium 137.7 mmol/L; chloride 106.2 mmol/L; calcium 2.30 mmol/L; phosphorus 1.70 mmol/L; carbon dioxide combining power 20.3 mmol/L; urea 5.70 mmol/L; creatinine 46.0 μmol/L; cystatin C 2.1 mg/L; uric acid 276.4 μmol/L; TSH 5.29 μIU/mL; total T4 165.29 nmol/L; total T3 3.44 nmol/L; free T4 9.71 pmol/L; free T3 4.59 pmol/L while receiving levothyroxine.	Renal stress and thyroid dysfunction predated the current admission.
9 months, current admission	Fever for 1 day with increased urine leukocytes.	CRP 68.3 mg/L; urine WBC 405.60/μL; leukocyte esterase (++); protein (+).	Findings supported complicated UTI/acute pyelonephritis.
2025-05-05	Endocrine, renal, urinary-protein, and lipid reassessment.	TSH 9.18 μIU/mL; free T4 5.81 pmol/L; creatinine 40.0 μmol/L; cystatin C 1.66 mg/L; urinary *α*1-microglobulin 96.80 mg/L, microalbumin 70.70 mg/L, IgG 24.40 mg/L; triglycerides 2.57 mmol/L, HDL-C 0.55 mmol/L, ApoA1 0.65 g/L.	Hypothyroidism recurred after treatment interruption; renal and urinary injury markers required follow-up.
2025-05-08 to 2025-05-14	DMSA, VCUG, and DTPA studies completed.	DMSA showed right upper/lower pole focal defects but no numerical split function; VCUG showed bilateral grade IV VUR; DTPA ERPF was 180.33 mL/min left and 133.77 mL/min right (57.4%/42.6%).	High-grade reflux with early right renal involvement was documented.
2025-05-19 to 2025-05-23	Planned admission for phimosis surgery; an afebrile seizure occurred during sleep before surgery.	No further seizure during observation after oxygen, monitoring, and phenobarbital sodium.	Surgery was postponed; neurologic follow-up and video-EEG were arranged.
Follow-up to 2025-08-26	Renal, endocrine, neurologic, and developmental reassessment.	No recurrent UTI; creatinine 25.0 μmol/L, cystatin C 1.39 mg/L, eGFR 103.66 mL/min/1.73 m^2^, urine protein/creatinine 0.12 g/g Cr; thyroid function normalized; video-EEG non-diagnostic; seven brief ocular-convergence episodes.	Renal indices improved, but seizure-like events and developmental delay required continued follow-up; repeat DMSA/VCUG were not performed.

On 23 February 2025, he had been hospitalized elsewhere for urinary tract infection complicated by sepsis, myocardial injury, bilateral hydronephrosis with ureteral dilatation, hypothyroidism, oral candidiasis, anemia, and abnormal brain development. Available results included WBC 21.46 × 10⁹/L, neutrophils 64.7%, lymphocytes 23.1%, hemoglobin 108 g/L, platelets 332 × 10⁹/L, CRP 120.6 mg/L, urine protein (+), leukocyte esterase (++), RBC 7.80/μL, WBC 512.67/μL, WBC clusters 28.90/μL, lactate 7.4 mmol/L, procalcitonin 10.2 ng/mL, creatinine 46.0 μmol/L, and cystatin C 2.1 mg/L. While receiving levothyroxine, TSH was 5.29 μIU/mL, total T4 165.29 nmol/L, total T3 3.44 nmol/L, free T4 9.71 pmol/L, and free T3 4.59 pmol/L. He improved after meropenem, piperacillin/tazobactam, and levothyroxine, but the family discontinued levothyroxine in early April.

The patient was the third child of non-consanguineous parents. He was delivered by cesarean section at 35 + 5 weeks, weighed 2,700 g, and had Apgar scores of 10 at 1 and 5 min. He was not admitted to the NICU and did not require respiratory support; there was no perinatal asphyxia, respiratory failure, heart failure, or birth trauma. Newborn heel-prick screening was reported as suspicious positive. During the neonatal period, TSH was 8.58 μIU/mL (reference 0.34–5.6 μIU/mL) and free T4 was 5.71 pmol/L (reference 7.86–14.41 pmol/L); congenital hypothyroidism was diagnosed and levothyroxine was started promptly. No definite infection was identified during the neonatal period. There was no known family history of genetic disease.

Developmentally, he achieved head control at 3 months but at 9 months could not sit steadily or crawl, and language and fine motor development were delayed. On admission, temperature was 37.4 °C, pulse 122/min, respiration 32/min, body weight 10.8 kg, length 71 cm, head circumference approximately 45.3 cm, and body mass index 21.4 kg/m^2^. Physical examination showed early anterior fontanel closure, a square face, mildly depressed nasal bridge, left strabismus, mild eversion of the upper lip, bilateral brachydactyly, generalized hypotonia, short penis, phimosis, and mild meatal congestion. Cardiopulmonary and abdominal examinations were unremarkable, and meningeal signs were negative (Figure 1).

**Figure 1:**
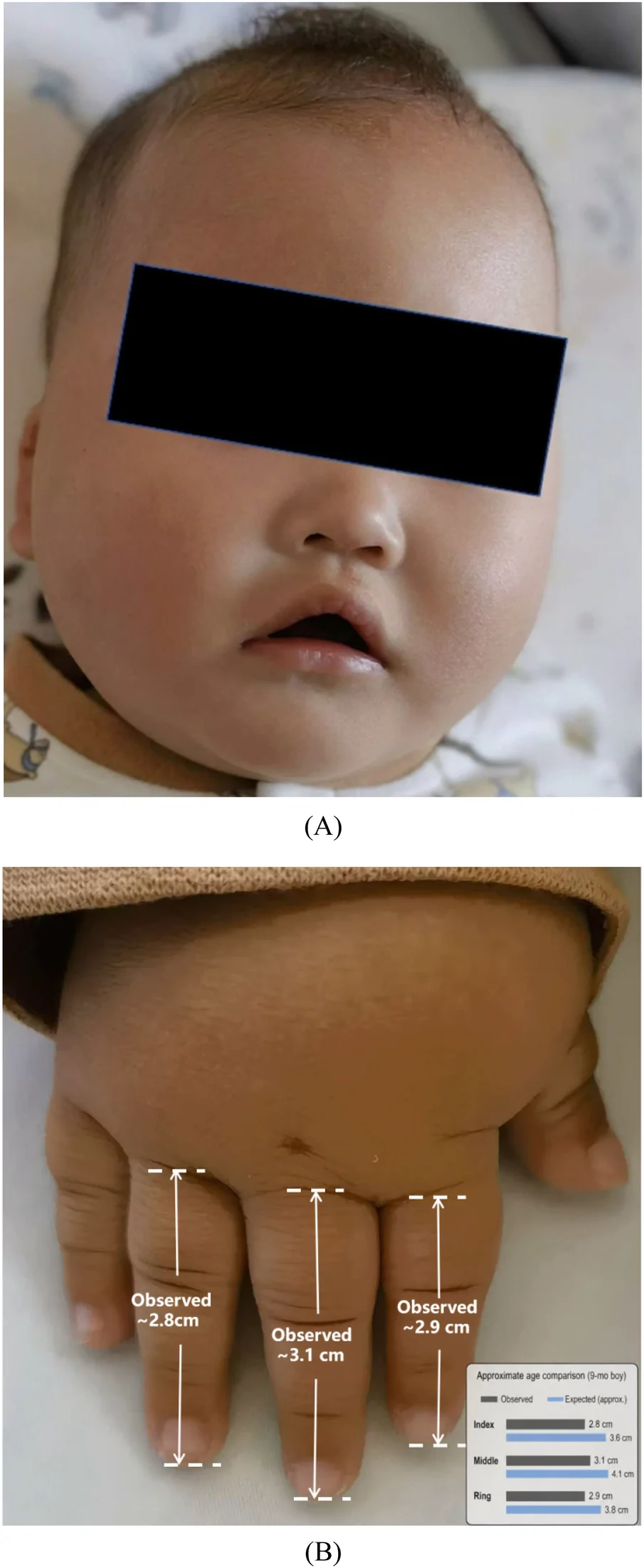
Clinical photographs of the patient. **(A)** Facial features showing a square face, mildly depressed nasal bridge, and mild eversion of the upper lip; the eyes are masked to protect privacy. **(B)** Hand photograph showing brachydactyly.

### 2.2 Diagnostic assessment

Admission laboratory results supported complicated urinary tract infection/acute pyelonephritis: WBC 9.60 × 10⁹/L with 70.0% neutrophils, hemoglobin 112 g/L, platelets 245 × 10⁹/L, and CRP 68.3 mg/L. Urinalysis showed RBC 21.10/μL, WBC 405.60/μL, leukocyte esterase (++), and protein (+). Serum creatinine was 40.0 μmol/L and cystatin C was 1.66 mg/L. Urinary *α*1-microglobulin was 96.80 mg/L (reference 0–12.5 mg/L), microalbumin 70.70 mg/L (reference 0–19 mg/L), and IgG 24.40 mg/L (reference 0–8 mg/L). The elevated cystatin C despite relatively preserved creatinine may indicate early renal dysfunction or acute renal stress in the setting of pyelonephritis and high-grade VUR, but a single value during acute illness requires cautious interpretation. Triglycerides were 2.57 mmol/L, HDL-C 0.55 mmol/L (reference 1.25–1.55 mmol/L), and ApoA1 0.65 g/L (reference 1.0–1.6 g/L). Immunologic screening showed IgA 0.18 g/L, IgG 6.39 g/L, and IgM 1.27 g/L; lymphocyte subsets did not show severe cellular immunodeficiency.

Renal ultrasonography showed bilateral hydronephrosis with ureteral dilatation; collecting-system separation measured approximately 11–12 mm on the left and 8–9 mm on the right, and ureteral diameters were approximately 6–7 mm. Voiding cystourethrography demonstrated bilateral grade IV VUR. Static 99mTc-DMSA scintigraphy showed focal sparse uptake defects in the upper and lower poles of the right kidney; the report did not provide numerical split renal function. Dynamic 99mTc-DTPA scintigraphy showed mildly delayed bilateral excretion and impaired right tubular secretory function. Effective renal plasma flow was 180.33 mL/min in the left kidney and 133.77 mL/min in the right kidney, corresponding to split function of 57.4% and 42.6%, respectively (Figure 2). Brain MRI showed bilateral lateral ventriculomegaly, a widened cisterna magna, and mildly widened left temporal subarachnoid space. Echocardiography showed a patent foramen ovale, and electrocardiography suggested sinus bradycardia with possible early repolarization. After levothyroxine interruption, TSH was 9.18 μIU/mL and free T4 was 5.81 pmol/L. Gesell assessment at age 9.2 months yielded an equivalent age of 6.6 months and a developmental quotient of 72, consistent with mild global developmental delay.

**Figure 2:**
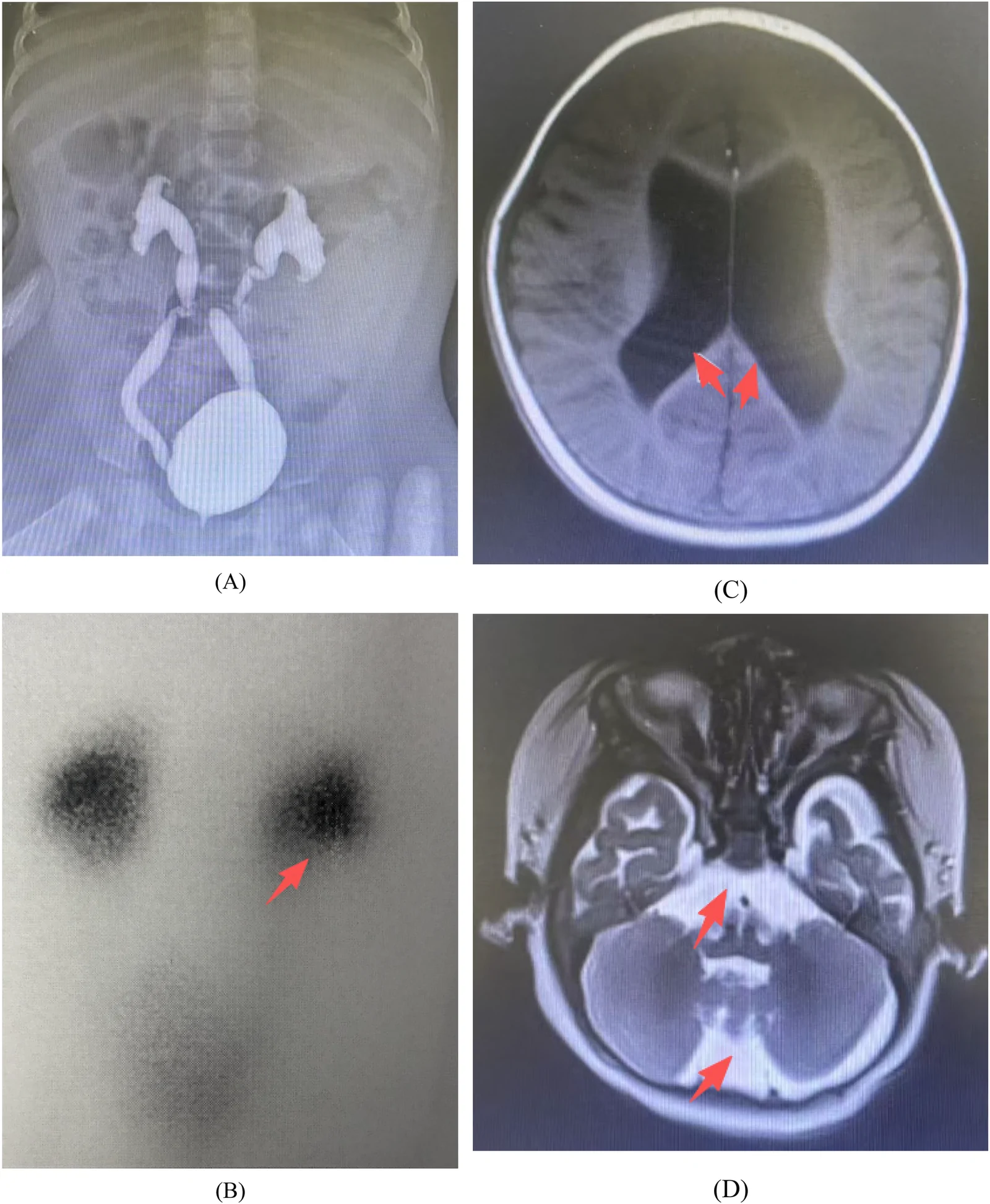
Imaging findings. **(A)** Voiding cystourethrography showing bilateral high-grade vesicoureteral reflux. **(B)** DMSA renal scintigraphy showing focal decreased uptake in the right kidney. **(C,D)** Brain MRI showing bilateral lateral ventriculomegaly, widened cisterna magna, and widened left temporal subarachnoid space.

Before genetic testing, the combination of developmental delay, hypotonia, dysmorphic features, brachydactyly, genitourinary anomalies, thyroid dysfunction, and multisystem involvement prompted consideration of syndromic neurodevelopmental disorders. Cornelia de Lange syndrome and Mowat-Wilson syndrome were among the phenotypic differentials, but no single clinical diagnosis accounted for the complete pattern. Broad genetic testing with CNV detection was therefore selected rather than a single-gene-only approach.

### 2.3 Genetic and laboratory investigations

Trio whole-exome sequencing (WES) was performed by an external clinical genetics laboratory with WES-based CNV analysis and parental comparison. Variants and CNVs were evaluated against population and disease databases and interpreted using ACMG/AMP and ACMG/ClinGen principles (9, 10). The laboratory report did not specify the library-preparation kit, sequencing-platform model, mean coverage, or CNV-calling software; these details were not inferred. Routine thyroid, renal, inflammatory, immunologic, and biochemical tests were performed as part of clinical care in the respective hospital laboratories. Assay-specific analyzer and reagent information was not recoverable from the retrospective records.

WES-based CNV analysis identified a *de novo* heterozygous 17p11.2 deletion, chr17:16,692,403–20,321,263, approximately 3.63 Mb in size. The interval includes RAI1 and the SMS critical region, and neither parent carried the deletion. Under ACMG/AMP sequence-variant and ACMG/ClinGen CNV standards, the deletion was classified as pathogenic and established the diagnosis of SMS (9, 10). Sequence analysis also identified a maternally inherited heterozygous DUOX2 variant, c.2635G > A (p.Glu879Lys), classified as likely pathogenic, and two heterozygous variants of uncertain significance: CCBE1 c.899G > T (p.Gly300Val), inherited from the mother, and RAG2 c.376G > C (p.Glu126Gln), inherited from the father. These variants do not explain the core SMS phenotype. The possible contribution of DUOX2 to hypothyroidism is unproven; no functional validation was performed, and an incidental clinically silent carrier state cannot be excluded (Table 2).

**Table 2:** Genetic findings and clinical interpretation.

Gene/region	Variant	Zygosity/type	Origin	Classification	Clinical interpretation
17p11.2 region	chr17:16,692,403-20,321,263; approx. 3.63 Mb deletion	Heterozygous CNV deletion	*De novo*	Pathogenic CNV	Includes RAI1 and the SMS critical region; establishes the diagnosis of SMS.
DUOX2	c.2635G > A; p.Glu879Lys	Heterozygous SNV	Maternal	Likely pathogenic	Possible but unproven contributor; maternally inherited and not functionally validated. An incidental clinically silent carrier state cannot be excluded.
CCBE1	c.899G > T; p.Gly300Val	Heterozygous SNV	Maternal	VUS	No direct explanatory role in the SMS phenotype; manage as a variant of uncertain significance.
RAG2	c.376G > C; p.Glu126Gln	Heterozygous SNV	Paternal	VUS	Does not explain the SMS phenotype; consider immune follow-up if clinically indicated.

### 2.4 Therapeutic intervention

The acute infection was treated with cefoperazone/sulbactam; after defervescence and urinalysis improvement, treatment was changed to oral amoxicillin/clavulanate. Levothyroxine was resumed. Because of bilateral grade IV VUR, recurrent febrile urinary tract infection, and renal scintigraphic abnormalities, prophylaxis was prescribed as amoxicillin/clavulanate potassium 76 mg orally once daily at bedtime. Pediatric urology planned reassessment for anti-reflux intervention. A definitive choice between endoscopic injection and ureteral reimplantation had not been made and would depend on recurrent febrile infection, reflux severity, renal damage, and subsequent clinical course. During a later admission for planned phimosis surgery, an afebrile sleep-related seizure was treated with oxygen, monitoring, and phenobarbital sodium; surgery was postponed.

### 2.5 Follow-up and outcomes

During the available follow-up, no recurrent urinary tract infection was documented. Renal testing improved to urea 2.70 mmol/L, creatinine 25.0 μmol/L, cystatin C 1.39 mg/L, eGFR 103.66 mL/min/1.73 m^2^, and urine protein/creatinine ratio 0.12 g/g Cr. A reliable standardized blood-pressure measurement was not available for percentile interpretation. TSH and free T4 returned to the normal range after levothyroxine was resumed. Repeat DMSA and VCUG were not performed. Video-EEG showed no definite abnormality. On 26 August 2025, seven brief episodes of ocular convergence, each lasting several seconds and resolving spontaneously, were observed and were considered possible seizure-like events rather than proof of epilepsy. Height and weight growth were satisfactory, whereas gross-motor, fine-motor, and language development remained delayed. Multidisciplinary urology, neurology, rehabilitation, endocrine, and genetic follow-up continued.

## 3 Discussion

### 3.1 Early recognition and diagnostic reasoning

The diagnostic value of this case lies in the convergence of findings before the classic SMS behavioral phenotype was fully expressed. Global developmental delay, hypotonia, brachydactyly, a square face, depressed nasal bridge, eversion of the upper lip, strabismus, early fontanel closure, genitourinary anomalies, hypothyroidism, and brain imaging abnormalities formed a syndromic pattern. Cornelia de Lange syndrome, Mowat-Wilson syndrome, and other syndromic neurodevelopmental disorders can overlap in infancy, but the combination of multisystem congenital findings supported broad genomic testing with CNV detection rather than a single-gene-only approach (1, 3, 4).

The 3.63-Mb *de novo* deletion includes RAI1 and adjacent genes, providing a parsimonious explanation for the early multisystem presentation. This is consistent with genotype-phenotype studies in which deletion carriers show more structural and organ-system abnormalities than individuals with intragenic RAI1 variants (1, 4, 7). The maternally inherited DUOX2, CCBE1, and RAG2 findings therefore require interpretation in the context of the primary pathogenic CNV rather than as competing explanations for the overall phenotype.

This case also illustrates the practical value of CNV detection. Microdeletions may be missed by routine karyotyping or by exome workflows restricted to single-nucleotide variants. Chromosomal microarray is a first-tier test for developmental disability or congenital anomalies, and exome-based CNV analysis can increase diagnostic yield (11, 12). Here, trio WES with CNV analysis and parental comparison identified the causal lesion while avoiding over-attribution of incidental heterozygous variants.

### 3.2 Bilateral high-grade VUR and renal risk

Genitourinary anomalies occur in a minority of individuals with 17p11.2 deletions, but the literature includes diverse renal, ureterovesical, and genital findings rather than a well-defined VUR phenotype (1, 7, 13). Table 3 compares the principal reported observations. The 2002 case of bilateral grade IV VUR is the closest comparator, whereas the present case adds infant presentation, quantitative DTPA split function, focal DMSA abnormalities, urinary injury markers, and short-term renal follow-up (8).

**Table 3:** Comparison of reported genitourinary involvement in smith-magenis syndrome.

Source	Design/sample	Molecular finding	Genitourinary finding	Relevance to present case
GeneReviews (1)	Synthesis of reported cases	17p11.2 deletion vs. RAI1 variant	Anomalies in 15%–35% of deletion carriers; collecting-system duplication, renal agenesis/ectopia, and ureterovesical obstruction or malposition.	Defines the deletion-associated genitourinary spectrum but not the frequency of VUR.
Girirajan et al. (7)	Genotype-phenotype cohort (*n* = 31)	17p11.2 deletion or RAI1 variant	Renal/urinary abnormalities in 15% of deletion carriers and 0/11 individuals with RAI1 variants.	Supports a contribution from the contiguous deletion rather than RAI1 alone.
Chou et al. (8)	Single case; 8-year-old boy	SMS with 17p11.2 deletion	Bilateral grade IV VUR requiring bilateral ureteroplasty.	Closest published VUR comparator.
Zhang et al. (13)	Prenatal/neonatal case	*De novo* 17p11.2p12 microdeletion	External genital defects.	Shows that genitourinary involvement can be evident early.
Present case	Single case; 9-month-old boy	*De novo* 3.63-Mb 17p11.2 deletion	Bilateral grade IV VUR, hydronephrosis/ureteral dilatation, phimosis, short penis, and early right renal involvement.	Adds quantitative renal scintigraphy and short-term follow-up.

VUR is a genetically heterogeneous congenital anomaly involving abnormal development or function of the ureterovesical junction, and both monogenic variants and CNVs contribute to CAKUT (14–16). Direct evidence linking RAI1 or another individual gene within the SMS interval to VUR is insufficient. Thus, SMS-related contiguous gene deletion may increase susceptibility to CAKUT/VUR, rather than VUR being attributed solely to RAI1 haploinsufficiency.

This patient has several risk factors for reflux nephropathy: bilateral grade IV reflux, recurrent febrile infection, hydronephrosis and ureteral dilatation, focal right DMSA defects, lower right split function, and abnormal urinary injury markers. Guidelines support individualized management based on reflux grade, infection recurrence, renal damage, renal growth, and bladder-bowel factors (17–19). Continuous antibiotic prophylaxis is supported for selected high-risk children and infants with grade III-V VUR, while adverse events and antimicrobial resistance must be monitored (20, 21). In this patient, amoxicillin/clavulanate prophylaxis was selected and no breakthrough UTI was documented during the short follow-up. This observation is reassuring but is too brief to determine long-term efficacy. Breakthrough febrile infection, progressive hydronephrosis, impaired renal growth, proteinuria, or new scarring would strengthen the indication for intervention. The definitive choice between endoscopic injection and ureteral reimplantation remains a pediatric-urology decision based on the subsequent renal and infection course.

The improvement in creatinine and cystatin C and the absence of recurrent UTI during available follow-up are reassuring but do not eliminate long-term risk. The urine protein/creatinine ratio was 0.12 g/g Cr, whereas standardized blood-pressure data and repeat DMSA/VCUG were unavailable. The discordance between cystatin C and creatinine during acute illness may have reflected early renal stress, but longitudinal measurement is more informative than either isolated value. Continued surveillance should include febrile urine cultures, urinalysis, proteinuria, renal function, blood pressure, renal growth, and urologic imaging when clinically indicated (17–22).

### 3.3 Hypothyroidism and cautious interpretation of DUOX2

The suspicious newborn screen, neonatal TSH 8.58 μIU/mL and free T4 5.71 pmol/L, and subsequent response to levothyroxine establish a clinically important thyroid chronology. After treatment interruption, TSH again increased and free T4 decreased; both normalized after replacement resumed. Because untreated hypothyroidism can worsen neurodevelopment during infancy, developmental delay should not be attributed to SMS alone, and sustained biochemical monitoring and dose adjustment remain essential (23). The normalization of thyroid indices after treatment strengthens the clinical rationale for continued replacement but does not resolve the genetic contribution to the original thyroid abnormality.

DUOX2 variants can cause thyroid dyshormonogenesis, but this heterozygous variant was inherited from the mother and was not functionally tested (24, 25). The available maternal information did not establish overt hypothyroidism. Therefore, the variant should not be presented as an established modifier or as a stand-alone explanation. It is a possible but unproven contributor, and an incidental clinically silent carrier state cannot be excluded. Clinical management should be guided by the child's thyroid course rather than by the modifier hypothesis.

### 3.4 Seizure and neurodevelopmental follow-up

Seizures occur in a minority of individuals with SMS, but this patient's evidence does not establish epilepsy (1, 26). He had one afebrile sleep-related seizure and later brief ocular-convergence episodes, while video-EEG showed no definite abnormality. Experimental data linking RAI1 deficiency to hippocampal excitability provide biological context but cannot establish the cause of this child's events (27). The normal video-EEG does not exclude intermittent events, but neither the imaging findings nor the observed episodes justify a definitive epilepsy diagnosis. Continued neurologic follow-up is appropriate, with the events described as possible seizure-like episodes rather than SMS-associated epilepsy.

Neurodevelopmental intervention should not wait for the later behavioral phenotype. Although height and weight growth were satisfactory, gross-motor, fine-motor, and language delays persisted. Rehabilitation, hearing and vision assessment, and serial measurement of motor, language, cognition, sleep, and behavior are therefore central to follow-up, alongside control of infection and hypothyroidism.

### 3.5 Strengths and limitations

Strengths of this report include diagnosis during infancy, integration of renal imaging with quantitative DTPA data, clarification of the thyroid chronology, and short-term renal, endocrine, neurologic, and developmental follow-up. The case also shows how a syndromic diagnosis changes care from episodic infection treatment to coordinated renal protection and developmental surveillance.

Limitations include the observational nature of a single case, limited follow-up, incomplete retrospective assay and treatment details, absence of numerical DMSA split function, lack of standardized follow-up blood-pressure percentiles and repeat DMSA/VCUG, and no functional validation of DUOX2. The relationship between the seizure-like events and SMS-associated epilepsy remains uncertain despite a non-diagnostic video-EEG. These gaps limit causal and prognostic inference and require longer longitudinal follow-up. The report was structured according to CARE case-report principles (28, 29).

## 4 Patient perspective

The patient was an infant and could not provide a personal perspective. His legal guardians participated in counseling about the genetic diagnosis and its renal, endocrine, neurologic, and developmental implications. They discussed antimicrobial prophylaxis, possible anti-reflux and phimosis procedures, levothyroxine treatment, rehabilitation, and the need for multidisciplinary follow-up, and provided written consent for publication of de-identified clinical information and images. Subjective emotional experiences were not prospectively recorded and are therefore not inferred.

## 5 Conclusion

SMS should be considered when an infant with developmental delay and hypotonia also has dysmorphic, skeletal, genitourinary, and endocrine findings; genomic testing should be capable of detecting CNVs. In this patient, the diagnosis linked bilateral high-grade VUR and early renal injury to a broader multisystem surveillance plan. The principal implication is the need for coordinated renal protection, thyroid replacement, cautious neurologic assessment, developmental rehabilitation, urologic decision-making, and genetic counseling.

## Data Availability

The datasets presented in this study can be found in online repositories. The names of the repository/repositories and accession number(s) can be found in the article/Supplementary Material.
